# Nucleus of the solitary tract A2 neurons control feeding behaviors via projections to the paraventricular hypothalamus

**DOI:** 10.1038/s41386-022-01448-5

**Published:** 2022-09-16

**Authors:** Stephanie Murphy, Metika Collis Glynn, Tiarani N. Dixon, Harvey J. Grill, Gavan P. McNally, Zhi Yi Ong

**Affiliations:** 1 School of Psychology, University of New South Wales, UNSW, Sydney, NSW 2052 Australia; 2 Department of Psychology, Institute of Diabetes Obesity and Metabolism, University of Pennsylvania, Philadelphia, PA 19104 USA

**Keywords:** Hypothalamus, Motivation

## Abstract

Hindbrain NTS neurons are highly attuned to internal physiological and external environmental factors that contribute to the control of food intake but the relevant neural phenotypes and pathways remain elusive. Here, we investigated the role of NTS A2 neurons and their projections in the control of feeding behaviors. In male TH Cre rats, we first confirmed selective targeting of NTS A2 neurons and showed that chemogenetic stimulation of these neurons significantly suppressed dark cycle food intake, deprivation re-feed and high fat diet intake. Despite reducing intake, activation of NTS A2 neurons had no effect on food approach, anxiety-like behaviors, locomotor activity, blood glucose levels nor did it induce nausea/malaise, thus revealing a selective role for these neurons in the consummatory aspect of food intake control. Pathway-specific mapping and manipulation of NTS A2 neurons showed that these effects were mediated by NTS A2 neurons projecting to the paraventricular nucleus of the hypothalamus (PVH) because chemogenetic activation of these projections, but not projections to bed nucleus of the stria terminalis (BNST), reduced food intake. Cell-type specific analyses demonstrated that activation of NTS A2 neurons recruited both PVH oxytocin (OT)- and corticotropin-releasing factor (CRF)-expressing neurons, and plasma analyses showed increased plasma corticosterone following NTS A2 stimulation. While we also showed that chemogenetic inhibition of NTS A2 neurons attenuated the intake inhibitory effects of CCK, the specificity of transgene expression was low. Together, these findings showed that NTS A2 neurons are sufficient to control the consummatory aspects of feeding, regardless of energy status or food palatability and identified their projections to PVH, but not BNST, in food intake control.

## Introduction

Eating is a tightly regulated homeostatic process. Perturbations to the internal (e.g. gut signals, inflammation) and external (e.g. food cues, stressors) environment, can disrupt this highly controlled system to either drive or suppress feeding. Neurons in the hindbrain are attuned to changes in the internal and external milieu and have long been implicated in food intake control [1–3]. However, the mechanisms of hindbrain-mediated control of feeding behaviors, including the mediating neuronal phenotypes and their projections, are only partially understood.

Ahlskog and Hoebel (1973) first showed that lesioning the ventral noradrenergic bundle causes hyperphagia and body weight gain in rats [4]. This suggests that noradrenergic neurons of hindbrain origin (e.g. nucleus of the solitary tract [NTS; A2], rostral ventral lateral medulla [RVLM; A1/C1]) are necessary for food intake suppression and energy balance control. Follow up studies targeting NTS A2 neurons supported this hypothesis and showed that NTS dopamine beta hydroxylase (DBH) neural lesions prevent intake suppression by gut peptide cholecystokinin (CCK) [5] and lithium chloride (LiCl) [6]. These findings indicate that NTS A2 neurons are required for food intake inhibition. The significance of NTS A2 neurons in feeding control was further substantiated by more recent studies using genetic manipulation techniques in mice which showed that stimulation of NTS A2 neurons reduces food intake [7, 8]. Together, these findings support a role for NTS A2 neurons in the control of food intake suppression.

However, there is also evidence that NTS A2 neurons may have different, even opposing roles in feeding behaviors that depend, at least in part, on their specific projection profiles [9]. NTS A2 neurons have diverse ascending projections to key brain regions controlling feeding behaviors including the parabrachial nucleus (PBN), arcuate nucleus of the hypothalamus (Arc), paraventricular nucleus of the hypothalamus (PVH), and bed nucleus of the stria terminalis (BNST). Of interest are the projections to PVH and BNST given their strong A2 inputs. Previous studies using DBH lesions have provided some insights into their function, albeit conflicting, and not specific to A2 inputs: Lesioning PVH noradrenergic inputs attenuates the ability of gut peptide oleoethylamine (OEA) to suppress food intake [10], but also blocks glucoprivation-induced increases in food intake [11]. Lesioning BNST noradrenergic inputs, on the other hand, has no effect on yohimbine-induced intake suppression [12]. Given that PVH and BNST receive noradrenergic inputs from NTS, RVLM and locus coeruleus [13, 14], these contrasting findings could be due to the lack of specificity of noradrenergic source. Further complicating the understanding of NTS A2 function is the anatomical organization and collateralization of NTS A2 neural projections. Lesioning noradrenergic inputs to PVH eliminates DBH fiber expression in BNST, and vice versa [12, 15], suggesting extensive collateralization between noradrenergic projections to PVH and BNST. Together, these discrepancies highlight the need to target NTS A2 neurons and their specific projections to better understand the role of NTS A2 neurons in food intake control.

Here, we addressed these issues using chemogenetics in a validated transgenic TH Cre rat [16] that expresses Cre recombinase in NTS A2 neurons. Our findings demonstrated that activation of NTS A2 neurons selectively suppresses the consummatory aspect of feeding behaviors regardless of energy status or food palatability, and they do so via their projections to the PVH, but not BNST.

## Materials and methods

### Animals

Male TH Cre rats (SD-TH-Cre^tm1sage^) (*n* = 105, Sage Laboratories, Cambridge, United Kingdom) [16] and wild type Sprague Dawley rats (*n* = 11, Animal Resources Centre, Murdoch, Western Australia) were 300–400 g upon arrival and were housed in ventilated racks in a climate-controlled room on a 12 h:12 h light/dark cycle (lights off 7:00 pm). Animals were individually housed for home cage food intake experiments and group housed for experiments carried out outside the home cage. Chow and water were available *ad libitum*, unless otherwise specified. All experimental procedures were approved by the University of New South Wales Animal Care and Ethics Committee.

### Stereotaxic surgery

Stereotaxic surgeries were performed as previously described [17]. For tracing experiments, rats received 100 nL unilateral infusions of the retrograde tracer, fluorogold (Fluorochrome LLC, CO) to PVH or BNST while rats in the behavioral experiments received bilateral NTS infusions (200 nL/side) of AAV-hSyn-DIO-eGFP or AAV-hSyn-DIO-hM3Dq. Rats in the A2^NTS→PVH^ and A2^NTS→BNST^ groups were also implanted with 26 GA guide cannulae targeting the PVH or BNST respectively. See supplementary information for details on surgeries, infusion of viral vectors and stereotaxic coordinates. Rats without NTS viral expression and/or have misplaced cannula were excluded from the study.

### Validation of TH Cre rat model to target NTS A2 neurons

Previous validation of TH Cre rats focused primarily on midbrain VTA TH neurons [16, 18, 19]. Whether TH neurons in caudal NTS also express Cre recombinase was unknown. To verify expression of Cre on NTS TH/A2 neurons, TH Cre rats (*n* = 3) were bilaterally injected with AAV-hSyn-DIO-eGFP to caudal NTS. After 3 weeks, rats were transcardially perfused, as previously described [17]. NTS sections were cut and immunohistochemistry performed to label TH and eGFP. The number of TH- and eGFP-labelled cells were counted from each NTS section at the level of the area postrema (Bregma −13.68 to −14.40 mm).

To confirm chemogenetic activation of NTS A2 neurons, TH Cre rats infused with AAV-hSyn-DIO-hM3Dq to the NTS received intraperitoneal (IP) injections of Vehicle (5% DMSO, 95% saline; *n* = 3) or 1 mg/kg CNO (*n* = 3). After 90 min, rats were perfused and brains removed. NTS sections were cut and immunohistochemistry performed to label TH, mCherry (label for hM3Dq) and c-Fos. The number of c-Fos immunopositive cells were counted from NTS sections and compared between Vehicle and CNO treatments.

### Microinfusions

To activate A2^NTS→PVH^ and A2^NTS→BNST^ pathways, 100 nL/side of 3 mM CNO or Vehicle were microinfused to the PVH and BNST respectively, as previously described [17]. Microinfusions were performed 45 min prior to test. This duration and CNO dose were chosen based on [20, 21].

### Behavioral experiments

Experiments commenced at least three weeks after surgeries. Rats received IP injections of Vehicle (5% DMSO, 95% saline) or CNO (0.3 mg/kg, 1 mg/kg; RTI International, NIMH Code C-929) 45 min prior to test. This injection pre-treatment time was chosen based on [21]. Treatments were counterbalanced within subjects and separated by 2-3 days to prevent any drug carry over effects.

#### Dark cycle chow intake

Rats were acclimated to individual housing and IP injections one week before commencement of experiments. During test day, chow was removed 2 h prior to dark cycle onset. At dark onset, chow was returned and the amount of chow consumed was measured at 2 h and 14 h. Rats were also weighed before injections and at 14 h.

#### Deprivation re-feed

Chow was removed from individually housed rats 24 h prior to test. At dark onset, chow was returned and intake measured at 0.5 h, 1 h, 14 h.

#### High fat diet intake

Individually housed rats were habituated to 1 h access of high fat diet (SF00-219, Specialty Feeds, Western Australia) in the light cycle for 4 days. During test day, chow was removed 2 h prior to test. High fat diet was presented during mid-light cycle and the amount consumed within 1 h was recorded, after which high fat diet was replaced with chow.

#### Food approach

Rats were habituated to an open field where powdered high fat diet was available in the center. During test day, rats were placed in the open field with high fat diet for 15 min. Behaviors were video recorded. The number of food zone entries, amount of time spent in food zone and latency to approach were scored by an experimenter blinded to treatment allocations.

#### Pica

Pica, a well-established model for nausea/visceral malaise in rodents [22], involves the ingestion of non-nutritive substances (e.g. kaolin clay), which is thought to provide protection to the gastric mucosa against toxins and alleviate nausea/visceral malaise [23]. TH Cre (hM3Dq) rats (*n* = 10) were habituated to kaolin clay in their home cages for at least 3 days. During test days, the amount of chow and kaolin clay consumed was measured at 15 h post dark cycle onset. As a positive control, naïve wild type Sprague Dawley rats (*n* = 7) were injected with either Vehicle or 0.15 M LiCl (20 mL/kg).

#### Elevated plus maze test

Anxiety-like behaviors were measured using the elevated plus maze, which consists of two arms: closed and open. TH Cre hM3Dq rats were placed in the middle of the maze and allowed to freely explore both arms for 5 min. Test sessions were video recorded. Time spent in each arm was scored by an experimenter blinded to the experimental conditions.

### Blood glucose analysis

TH Cre hM3Dq rats were injected with Vehicle or 1 mg/kg CNO and blood was collected from tail tip onto glucose strips at 0 min, 30 min, and 60 min post-injection. Blood glucose levels were determined with a glucometer (Accu-Chek Performa, Roche).

### Plasma corticosterone analysis

TH Cre hM3Dq rats were administered 1 mg/kg CNO or Vehicle in the mid light cycle. After 60 min, blood was obtained through a tail nick, collected in an EDTA-coated microfuge tube and immediately placed on ice. Blood was centrifuged at 4 °C, 4000 rpm for 15 min. Plasma was isolated and kept in −20 °C until plasma corticosterone analysis, which was performed according to Corticosterone ELISA kit (Abcam) manufacturer’s instructions.

### Immunohistochemistry

Chromogenic immunohistochemistry was performed for anterograde tracing of NTS A2 neurons, OT/c-fos and CRF/c-fos. Fluorescent immunohistochemistry was performed for viral verification and retrograde tracing. See supplementary information for details.

### Statistical analysis

Data are expressed as mean ± SEM. All experiments were analyzed using a mixed model repeated measures ANOVA where within subject factors are treatment and time, while between subject factor is genotype. When ANOVA identified an interaction, data were split into genotype and analyzed with pairwise comparisons and Bonferroni correction for multiple comparisons.

## Results

### Validation of TH Cre rat model

**Fig. 1:**
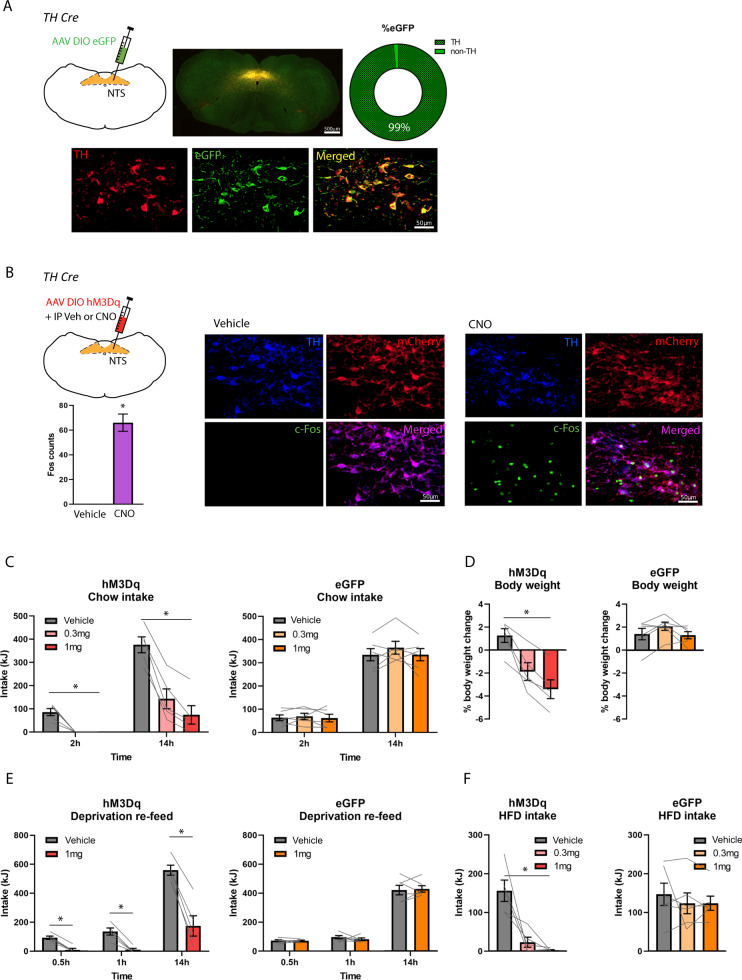
Validation of TH Cre rat model to target and manipulate NTS A2 neurons. **A** TH Cre rat injected with AAV DIO eGFP to the NTS. 99% of eGFP cells colocalise with TH-expressing neurons in the NTS. **B** Average number of c-Fos labelled cells per NTS section. CNO treatment in TH Cre rat injected with AAV DIO hM3Dq increased c-Fos expressing cells in the NTS, when compared to Vehicle treatment. **C** In hM3Dq rats, but not eGFP rats, CNO treatment (0.3 mg and 1 mg) significantly reduced chow intake at 2 h and 14 h post dark cycle onset, when compared to Vehicle treatment. **D** CNO treatment in hM3Dq rats, but not eGFP rats, reduced body weight, compared to Vehicle. **E** CNO (1 mg) significantly reduced chow intake in hM3Dq rats, but not eGFP rats after 24 h food deprivation at 0.5 h, 1 h and 14 h post dark cycle onset, when compared to Vehicle treatment. **F** CNO treatment (0.3 mg and 1 mg) significantly suppressed high fat diet intake in the light cycle in hM3Dq rats but not eGFP rats, when compared to Vehicle treatment. *P < 0.05.

Following infusions of AAV-DIO-eGFP to the NTS of TH Cre rats (*n* = 3), we showed that 99% of eGFP-expressing NTS cells were immunopositive for TH (Fig. 1A), thus confirming selective viral expression on NTS TH neurons. We also examined NTS hM3Dq expression and found 78% of hM3Dq-expressing NTS cells that expressed TH. We then showed that chemogenetic activation of NTS TH neurons (*n* = 3) increased the number of c-Fos-immunopositive neurons in the NTS, when compared to Vehicle treatment (*n* = 3) (*t* = −9.491, *P* < 0.05; Fig. 1B). Together, these data confirmed viral transfection of GFP and hM3Dq in NTS TH neurons and validated this TH Cre rat strain as a suitable rat model for targeting NTS TH/A2 neurons.

### Activation of NTS A2 neurons reduces chow intake and body weight

We examined whether activation of NTS A2 neurons reduces chow intake in the dark cycle when rats normally feed. Analysis of chow intake after Vehicle and CNO treatments in hM3Dq (*n* = 5) and eGFP (*n* = 6) rats at 2 h and 14 h post dark onset revealed a treatment x time x genotype (*F*_(2,18)_ = 41.592, *P* < 0.01) interaction. This indicates a differential effect of CNO on hM3Dq and eGFP rats. Bonferroni corrected multiple comparisons showed that both doses of CNO significantly reduced chow intake when compared to Vehicle (*P* < 0.01), with 0.3 mg CNO also significantly different from 1 mg CNO (*P* < 0.05; Fig. 1C) at 2 h and 14 h. For eGFP rats, there was no effect of treatment (*F*_(2,10)_ = 0.940, *P* > 0.05; Fig. 1C). Thus, CNO had no effect on chow intake in eGFP rats, indicating the specificity of CNO in hM3Dq rats.

When analyzed for body weight change, there was a treatment x genotype interaction (*F*_(2,18)_ = 21.327, *P* < 0.01). In hM3Dq rats, both doses of CNO significantly reduced body weight compared to Vehicle (*P* < 0.05; Fig. 1D). Consistent with the null effects on food intake in eGFP rats, there was also no effect of CNO on body weight (*F*_(2,10)_ = 2.198, *P* > 0.05; Fig. 1D).

### Activation of NTS A2 neurons reduces re-feed following food deprivation

We then asked whether activation of NTS A2 neurons suppresses chow intake in hungry rats who were food deprived for 24 h. We found a treatment x time x genotype (*F*_(2,18)_ = 32.054, *P* < 0.01) interaction on chow intake after food deprivation. Separate analyses were performed for hM3Dq and eGFP rats: In hM3Dq rats, CNO significantly suppressed chow intake at 0.5 h, 1 h and 14 h post dark onset (*P* < 0.01; Fig. 1E), but in eGFP rats, there was no effect of treatment on chow intake (*F*_(1,5)_ = 0.026, *P* > 0.05; Fig. 1E).

### Activation of NTS A2 neurons reduces high fat diet intake

We next examined the effects of NTS A2 neuron activation on high fat diet intake. Results showed a treatment x genotype interaction (F_(2,18)_ = 7.892, *P* < 0.01). Subsequent analysis showed that in hM3Dq rats, but not in eGFP rats, there was a main effect of treatment (*F*_(2,8)_ = 17.438, *P* < 0.01), where 1 mg CNO (*P* < 0.01) but not 0.3 mg CNO (*P* = 0.077) significantly reduced high fat diet intake (Fig. 1F).

### Activation of NTS A2 neurons reduces chow intake independent of nausea/malaise

**Fig. 2:**
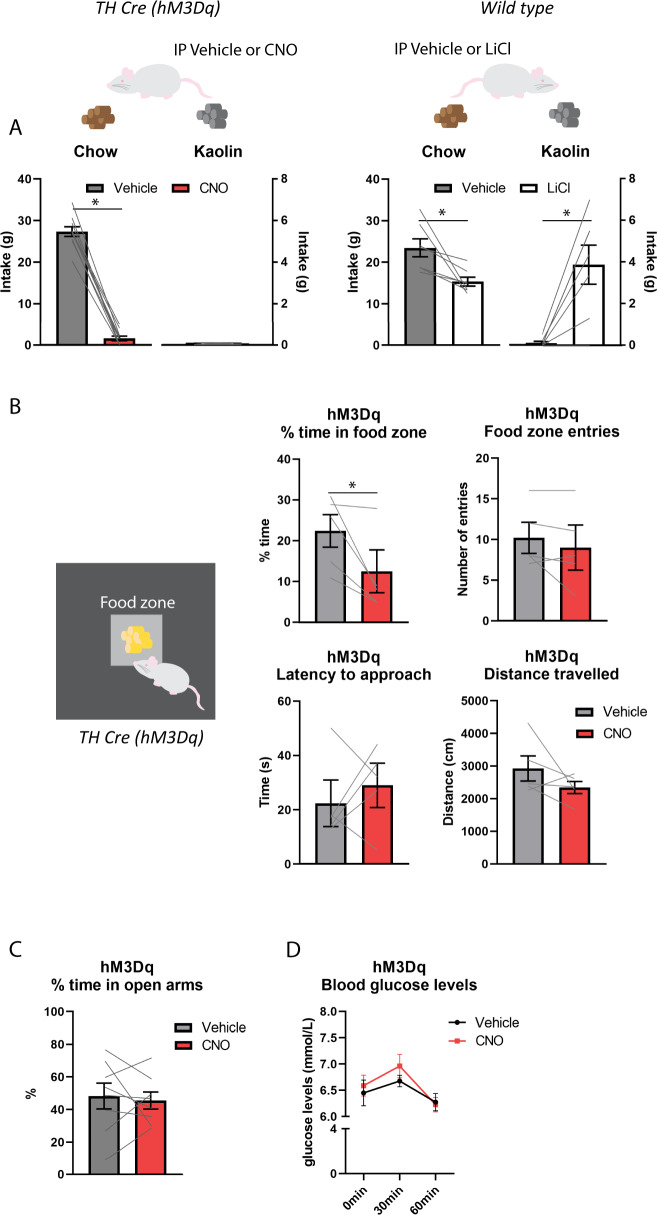
Effects of chemogenetic activation of NTS A2 neurons on kaolin intake, food approach, anxiety-like behaviors and blood glucose levels. **A** In hM3Dq rats, CNO treatment significantly suppressed chow intake without any effects on kaolin clay intake. As a positive control, wild type rats injected with 0.15 M LiCl significantly suppressed chow intake and increased kaolin clay intake. This indicates that NTS A2 neuron stimulation does not induce pica. **B** CNO treatment in hM3Dq rats reduced the amount of time spent in the food zone but had no effect on the number of food zone entries, latency to approach nor total distance travelled, suggesting that activation of NTS A2 neurons does not affect food approach behaviors or locomotor activity. **C** Elevated plus maze test was used to test anxiety-like behaviors. CNO treatment did not affect the percent time spent in the open arms, when compared to Vehicle treatment in hM3Dq rats. **D** Blood glucose levels did not differ between Vehicle or CNO treatment in hM3Dq rats. *P < 0.05.

To assess whether NTS A2 neuron activation also induces nausea/malaise, we examined pica by measuring kaolin clay intake. Results showed a treatment x food interaction (*F*_(1,9)_ = 455.618, *P* < 0.01) such that CNO significantly reduced chow intake (*t* = 21.285, *P* < 0.01) but had no effect on kaolin clay intake (*t* = 0.802, *P* > 0.05) when compared to Vehicle (Fig. 2A). As expected, LiCl reduced chow intake (*t* = 3.122, *P* < 0.05) and increased kaolin clay intake (*t* = −4.187, *P* < 0.01; Fig. 2A). Thus, as opposed to LiCl, stimulation of NTS A2 neurons did not induce nausea/malaise.

### Activation of NTS A2 neurons does not affect food approach behavior

Next, we determined whether, in addition to consumption, activation of NTS A2 neurons in hM3Dq rats also reduces food approach behavior. In an open field, high fat diet was placed in the middle and the number of ‘food zone’ entries and percent time spent in the ‘food zone’ were analyzed. Results showed that CNO significantly reduced percent time spent in food zone (*t* = 2.918, *P* < 0.05) but had no effect on the number of food zone entries (*t* = 1.177, *P* > 0.05), latency to enter food zone (ti = 0.71, *P* > 0.05) or total distance travelled (*t* = 1.385, *P* > 0.05; Fig. 2B), indicating that activation of NTS A2 neurons reduced high fat food intake without affecting food approach or overall activity.

### Activation of NTS A2 neurons has no effect on anxiety-like behaviors

We also examined whether NTS A2 neurons induce anxiety-like behaviors. Elevated plus maze test results showed no difference in the percent time spent in open arms (*t* = 0.368, *P* > 0.05; Fig. 2C) between Vehicle vs CNO treatment. Thus, consistent with previous studies [7, 24], activation of NTS A2 neurons did not induce anxiety-like behaviors.

### Activation of NTS A2 neurons has no effect on blood glucose levels

Given that NTS neurons are sensitive to changes in glucose levels [25**–**27] and that chronic lesioning of noradrenergic inputs to PVH results in elevated blood glucose levels [28], we examined whether activation of NTS A2 neurons altered blood glucose levels. Results show no main effect of treatment (*F*_(1,7)_ = 0.817, *P* > 0.05), thus indicating that acute activation of NTS A2 neurons had no effect on blood glucose levels (Fig. 2D).

### NTS A2 neural projections

**Fig. 3:**
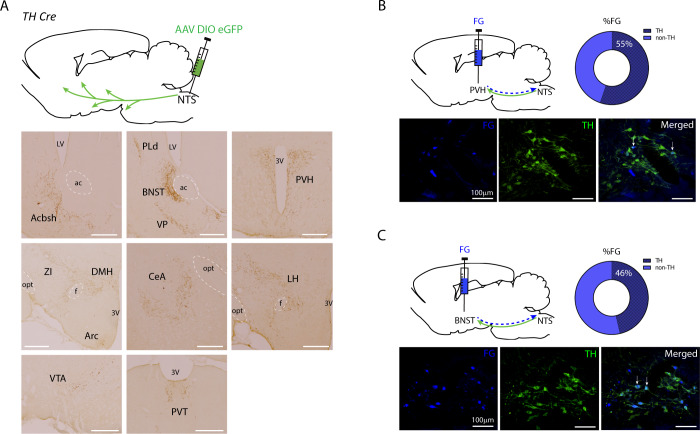
Anterograde and retrograde tracing of NTS A2 neurons. **A** Viral anterograde tracing of NTS A2 neurons show viral/GFP expression at the caudal nucleus accumbens shell (Acbsh), bed nucleus of the stria terminalis (BNST), paraventricular nucleus of the hypothalamus (PVH), dorsomedial hypothalamus (DMH), central nucleus of the amygdala (CeA), lateral hypothalamus (LH), ventral tegmental area (VTA) and paraventricular thalamus (PVT). Scale bars represent 200 µm. **B** Retrograde tracing of NTS A2 neurons to PVH using fluorogold (FG). 55% of FG expressing neurons in the NTS are TH positive. **C** Retrograde tracing of NTS A2 neurons to BNST using FG. 46% of BNST neurons are TH positive. White arrows indicate colocalization of FG and TH neurons.

To characterize NTS A2 neural projections, TH Cre rats (n = 3) were injected with AAV-DIO-eGFP to NTS and eGFP fiber expression mapped. Expression of eGFP was localized in brain regions implicated in feeding control including nucleus accumbens shell, BNST, PVH, dorsomedial hypothalamus, Arc, lateral hypothalamus, central amygdala, paraventricular thalamus and the ventral tegmental area (Fig. 3A). Retrograde tracing with FG injected into the PVH or BNST further confirmed ~50% of PVH or BNST-projecting neurons are TH positive (Fig. 3B, C, Supplementary Table 1).

### Activation of A2^NTS→PVH^ neurons reduces chow intake

**Fig. 4:**
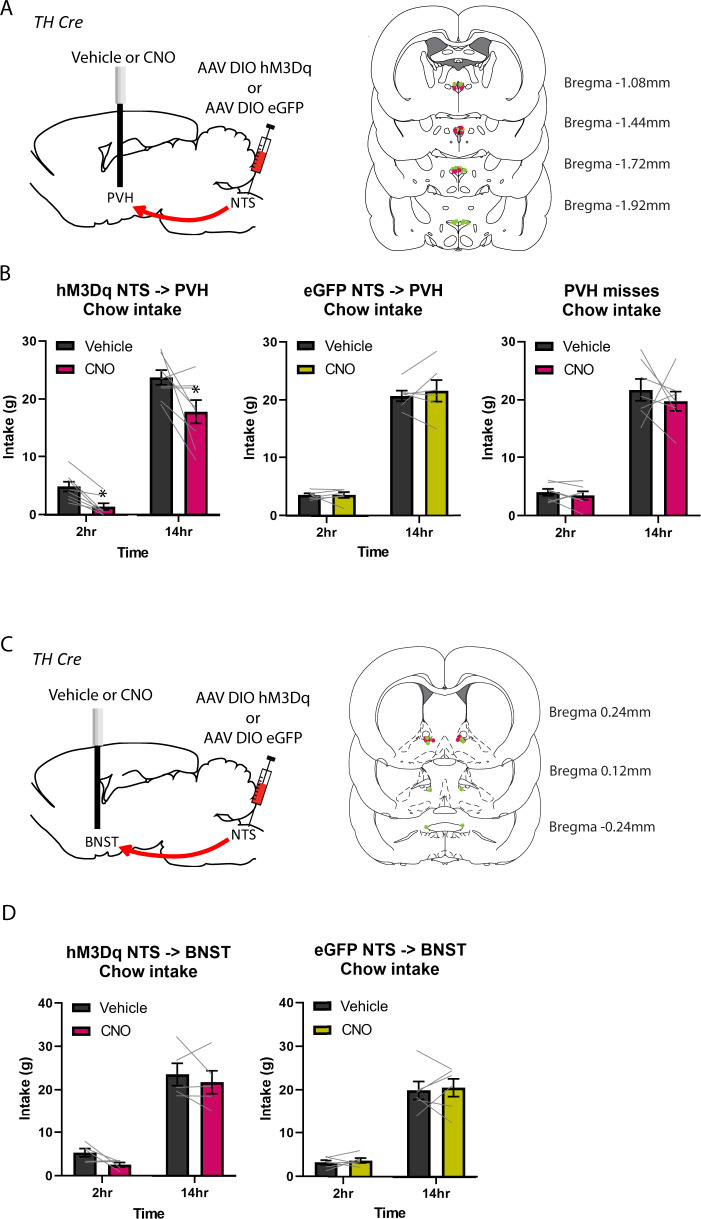
Pathway specific stimulation of NTS A2 neurons. **A** Schematic diagram of virus injections at the NTS and cannula implantation at the PVH. PVH cannula placements indicated in magenta (hM3Dq group) and green (eGFP group). **B** CNO activation of A2^NTS→PVH^ neurons significantly suppressed chow intake at 2 h and 14 h in hM3Dq rats but not in eGFP rats. **C** Schematic diagram of virus injections at the NTS and cannula implantation at the BNST. BNST cannula placements indicated in magenta (hM3Dq group) and green (eGFP group). **D** CNO activation of A2^NTS→BNST^ neurons had no effect on chow intake. *P < 0.05.

To examine whether activation of A2^NTS→PVH^ neurons reduces dark cycle chow intake, we infused Vehicle or CNO to PVH of hM3Dq (*n* = 8) and eGFP (*n* = 5) rats (Fig. 4A). Results showed a treatment x genotype interaction (*F*_(1,11)_ = 7.571, *P* < 0.05). Follow up analyses showed that in hM3Dq rats, CNO significantly reduced chow intake at 2 h (*t* = 4.927, *P* < 0.01) and 14 h (*t* = 2.526, *P* < 0.05), compared to Vehicle (Fig. 4B), while in eGFP rats, there was no difference in chow intake between CNO or Vehicle at 2 h (t = −0.093, *P* > 0.05) and 14 h (*t* = 0.051, *P* > 0.05). Thus, selective activation of A2^NTS→PVH^ neurons reduced chow intake.

### Activation of A2^NTS→BNST^ neurons has no effect on chow intake

We then examined whether chemogenetic activation of A2^NTS→BNST^ neurons controls food intake. Vehicle or CNO was infused to the BNST of hM3Dq (*n* = 5) and eGFP (*n* = 6) rats (Fig. 4C). There was no main effect of treatment (*F*_(1,9)_ = 0.699, *P* > 0.05), treatment x genotype (*F*_(1,9)_ = 1.920, *P* > 0.05), treatment x time (*F*_(1,9)_ = 0.177, *P* > 0.05), or treatment x time x genotype interaction (*F*_(1,9)_ = 0.126, *P* > 0.05), indicating that A2^NTS→BNST^ neurons do not control food intake (Fig. 4D).

### Activation of NTS A2 neurons increases c-Fos in PVH OT and CRF neurons

**Fig. 5:**
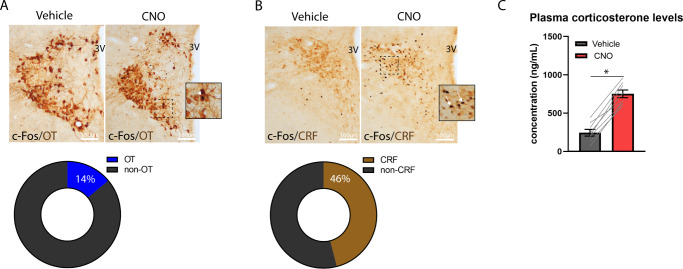
Phenotypes of PVH neurons activated by NTS A2 neuron stimulation. TH Cre rats were injected with AAV DIO hM3Dq to the NTS and injected with Vehicle or CNO. **A** Following CNO treatment, 14% of c-Fos expressing cells are OT positive. **B** Following CNO treatment, 46% of c-Fos expressing cells are CRF positive. **C** CNO treatment in hM3Dq rats significantly elevated plasma corticosterone levels, when compared to Vehicle treatment. White arrows indicate colocalization of c-Fos and OT or CRF neurons. *P < 0.05.

To determine the PVH neural phenotypes activated by NTS A2 neurons, TH Cre rats injected with AAV-DIO-hM3Dq to the NTS received either IP CNO (*n* = 4) or Vehicle (*n* = 4) and immunohistochemistry performed on 2 series of PVH sections. The first series labelled OT and c-Fos, while the second series labelled CRF and c-Fos. Total c-Fos counts were averaged between the first and second series. While activation of NTS A2 neurons showed higher number of c-Fos labelled PVH neurons, this was not statistically significant when compared to Vehicle treatment (*t* = −1.155, *P* > 0.05). Nonetheless, further analysis into the phenotypes of cells activated by NTS A2 neurons revealed that, OT and CRF neurons constitute 14% and 46% of c-Fos neurons, respectively (Fig. 5A, B), while 40% were unlabelled c-Fos cells. These proportions of activated OT and CRF cells were significantly higher than that in Vehicle treatment (OT: *t* = −3.44, *P* < 0.05; CRF: *t* = -2.71, *P* < 0.05; Supplementary Table 2). Hence, chemogenetic stimulation of NTS A2 neurons activates both PVH OT and CRF neurons, with a higher percentage of CRF neurons being activated.

### Activation of NTS A2 neurons increases plasma corticosterone levels

Consistent with increased activation of PVH CRF neurons, CNO significantly increased plasma corticosterone levels (*t* = −12.626; *P* < 0.01; Fig. 5C), compared to Vehicle.

## Discussion

We investigated the role of NTS A2 neurons in the control of feeding behaviors and identified both anatomical and behavioral mechanisms mediating their intake inhibitory effects. We demonstrated for the first time, in a TH Cre rat, that chemogenetic activation of NTS A2 neurons reduced food intake irrespective of energy status and food palatability. We also showed no effect on food approach, locomotor activity, anxiety-like behaviors, malaise or blood glucose levels, therefore indicating that NTS A2 neurons suppress food intake independent of these factors. Using pathway specific manipulations of NTS A2 neurons, we found that A2^NTS→PVH^ neurons, but not A2^NTS→BNST^ neurons, reduced chow intake. Further analysis into the PVH cell types activated by NTS A2 neurons revealed that OT and CRF neurons were recruited, with stronger recruitment of the latter. Taken together, these findings identify NTS A2 neurons as important regulators of food intake suppression and A2^NTS→PVH^ neurons as a pathway mediating the intake suppressive effects of NTS A2 neurons.

### Activation of NTS A2 neurons suppresses food intake and body weight without causing malaise

Previously, investigations into the role of NTS A2 neurons in regulating food intake in the rat, relied on saporin toxin lesions which cause permanent loss of NTS A2 neurons. More recent studies in mice utilized transgenic TH Cre or DBH Cre animals to target and reversibly manipulate these neurons to examine their function. However, the ability to target NTS A2 neurons in a rat has previously been lacking. This is important given the advantages of rats over mice for the study of more complex cognitive processes and their interplay with reward and motivation [29]. Thus, transgenic rat models provide the flexibility to study more complex behaviors while enabling selective cell type manipulation. Here, we showed that a TH Cre rat can be used to target and manipulate NTS A2 neurons. Consistent with studies using transgenic TH Cre mice [7], our results showed that chemogenetic activation of NTS A2 neurons robustly suppressed chow intake. This intake suppression persisted throughout the dark cycle and was accompanied by significant reductions in body weight, an effect that was less prominent in mice [7]. Importantly, we extend these findings to show that NTS A2 neurons are also sufficient to suppress food intake after an overnight fast and high fat diet intake. Thus, NTS A2 neurons not only suppress food intake under normal feeding conditions but also the consumption of palatable foods beyond homeostatic need. Interestingly, despite consuming less high fat diet, rats still exhibited robust approach behaviors towards the food, identifying a clear distinction between the role of NTS A2 neurons in consummatory versus appetitive preparatory aspects of feeding behavior [30]. Furthermore, we also provide new evidence that the robust suppression of feeding behaviors by NTS A2 neurons is not secondary to visceral malaise/nausea. These findings are reminiscent of past work showing that NTS A2 neurons do not mediate the aversive effects of LiCl, as rats with NTS A2 neuron lesions are still able to condition a flavor avoidance to LiCl [6]. Since activation of NTS A2 neurons control both homeostatic and palatability-driven feeding behaviors without incurring aversive states, these neurons could represent a critical target for controlling overeating, which often stems from dysregulated physiological signaling and heightened sensitivity to food palatability and environmental cues [31, 32].

### Activation of NTS A2^NTS→PVH^ neurons suppresses food intake

We then examined the neural pathways through which NTS A2 neurons suppress food intake. Previous mouse studies revealed that NTS A2 neurons suppress food intake via their projections to the PBN [7]. Here, we extend these findings to show that targeted stimulation of A2^NTS→PVH^, but not A2^NTS→BNST^ neurons significantly suppressed chow intake in the dark cycle. This intake inhibitory role of A2^NTS→PVH^ supports previous work where rats with PVH noradrenergic lesions show impaired ability to suppress food intake and are prone to develop diet-induced obesity [5, 10, 28]. However, lesioning PVH noradrenergic inputs also attenuates glucoprivation-induced increases in food intake [11], indicating an intake stimulatory role of these inputs that is likely mediated via other noradrenergic sources. Indeed, RVLM A1/C1 neurons (which send projections to PVH) are a putative noradrenergic source for driving glucoprivic feeding as activation of RVLM A1/C1 neurons increases food intake and activates PVH neurons [20, 33]. Thus, distinct noradrenergic sources are likely to mediate different, and even opposing feeding behaviors. It is also interesting that independent targeting of PVH and BNST A2 pathways showed clear functional differences despite past evidence for collateralization [13, 14]. The role of axon collaterals is not well-defined but they are likely required for synchronicity of multiple responses (e.g. increased HPA axis stimulation, reduced food intake) by a specific stimulus (e.g. stress) [34]. Nonetheless, these pathway specific differences in function reflect the heterogeneity of NTS A2 neurons and highlight the importance of isolating individual circuits to examine function.

### Activation of NTS A2 neurons recruits PVH OT and CRF neurons

As activation of A2^NTS→PVH^ neurons suppressed food intake, we sought to identify PVH cell types activated by NTS A2 neuron stimulation. We showed that chemogenetic activation of NTS A2 neurons recruited OT and CRF neurons, with a greater recruitment of the latter. Whether NTS A2 neurons activate CRF and OT neurons directly or indirectly remains to be determined. Nonetheless, this finding supports previous studies where rats with PVH noradrenergic lesions showed limited OT and CRF neuron activation by stress and satiation signals [6, 10]. Additionally, PVH CRF and OT neurons are activated by prolactin releasing peptide (PrRP) [35, 36], a neuropeptide highly co-expressed in A2 neurons [37], suggesting possible recruitment of CRF and OT neurons via PrRP release. We also showed that 40% of the activated neurons were neither OT nor CRF neurons. Previous studies indicate that the appetite suppressing CCK ^NTS→PVH^ neurons come in close contact with PVH melanocortin-4 receptor (MC4R) neurons [38], which are implicated in food intake control [39]. While some MC4R-expressing neurons co-express OT or CRF, there is a subset of appetite regulatory MC4R neurons that do not [40], thus suggesting that MC4R neurons could also constitute a portion of PVH neurons activated by NTS A2 neurons.

Despite differences in recruitment of CRF and OT neurons following activation of NTS A2 neurons, there was no overall difference in the number of PVH cells activated. The finding was not entirely surprising given that NTS A2 neurons, which co-express multiple neuropeptides/neurotransmitters, including noradrenaline, PrRP [37] and glutamate [41], can either inhibit or stimulate neurons in the Arc, an effect which relies on both noradrenergic and glutamatergic signaling [9]. Furthermore, noradrenaline itself can either increase or decrease PVH cell excitability [42**–**44], depending on the cell type and adrenergic receptors stimulated.

### Activation of NTS A2 neurons increases plasma corticosterone levels

The finding that NTS A2 neurons predominantly activated PVH CRF neurons prompted us to examine whether the HPA axis was activated in response to NTS A2 neuron stimulation. Indeed, we showed that chemogenetic activation of NTS A2 neurons increased plasma corticosterone levels. This finding supports previous studies showing that NTS A2 neurons are recruited by psychogenic stressors [45], which can in turn activate the HPA axis. It may seem surprising that we did not observe anxiety-like behavior following stimulation of NTS A2 neurons. However, while stressors including footshock can evoke corticosterone release as a stress response, evidence for corticosterone driving anxiety-like behaviors and stress-induced behaviors is conflicting [46**–**48]. Thus, increased corticosterone following activation of NTS A2 neurons may not necessarily trigger anxiety-like behaviors. Corticosterone also increases metabolism, as well as learning and memory [49]. Although these functions are beyond the scope of our study, current evidence suggest that NTS noradrenergic neurons facilitate memory retention [50, 51]. Whether this is mediated through an increase in corticosterone release remains to be determined. In addition, corticosterone has been shown to participate in the feeding effects of glucagon-like peptide-1 (GLP-1) where blocking corticosterone release enhances the intake suppressive effects of GLP-1 [52]. This finding suggests that the neuroendocrine effects of GLP-1 counteract its intake inhibitory effects. The significance of this interaction and whether this interaction exists with NTS A2 neurons is unclear. Nonetheless, the robust suppression in food intake following stimulation of NTS A2 neurons observed in our study suggests that that effects of NTS A2 neurons are unlikely influenced by peripheral corticosterone.

We only used elevated plus maze to measure anxiety-like behaviors in rats. Other behavioral tests that could be used to measure anxiety include light-dark box or startle box. While our data on anxiety-like behaviors are consistent with previous studies in mice using open field test (i.e., no difference in time spent in the center of the open field with NTS A2 neuron activation) [7], performing the anxiety tests mentioned above would also be useful to confirm the effects of NTS A2 neuron activation on anxiety-like behaviors.

### CNO attenuates CCK-induced intake suppression in TH Cre rat expressing inhibitory designer receptor hM4Di in NTS

Given that our behavioral experiments only examined the sufficiency of NTS A2 neurons in feeding behaviors, we also wanted to determine whether these neurons are necessary to control food intake (See supplementary information). We found that chemogenetic inhibition of NTS A2 neurons significantly attenuated CCK-induced intake inhibition, which supports past lesion studies [5] and suggests that these neurons are physiologically relevant in food intake control. However, this finding has to be interpreted with caution as analysis of hM4Di/mcherry expression in the NTS showed only 32% of hM4Di/mcherry neurons express TH. The lack of hM4Di specificity could be explained by possible ectopic Cre expression, which was previously documented in TH Cre mice in the VTA [53]. Other possibilities include, low level of TH protein undetectable by immunohistochemistry or post-translational modification of TH mRNA [54]. If these possibilities were true, then the low selectivity of hM4Di would also be observed with other transgene expression. However, we showed 99%, 78% and 67% colocalization of the transgene with TH in the NTS when we used AAV5-hsyn-DIO-eGFP, AAV5-hsyn-DIO-hM3Dq and AAV9-hsyn-DIO-mcherry, respectively. This suggests that there is some degree of interaction between AAV serotype and the transgene that could have resulted in inconsistent recombination and transgene expression in the transgenic rat. These inconsistencies indicate that this TH Cre rat could be used to assess the anatomical distribution/projections of NTS TH neurons and sufficiency of these neurons in different behaviors/functions but its suitability for other applications (e.g., neural inhibition) requires further validation prior to experimentation.

In sum, we specifically targeted and activated rat NTS A2 neurons to determine their role in food intake control. We showed that activation of NTS A2 neurons suppresses both homeostatic and non-homeostatic feeding behaviors. Importantly, these effects were selective to intake and were not secondary to changes in food approach, locomotor activity, anxiety-like behaviors, nausea/malaise or blood glucose levels, thus identifying the NTS A2 neurons as an attractive target for controlling overeating. We also showed for the first time, projection-specific effects of NTS A2 neurons, such that projections to PVH, but not BNST, suppressed food intake. While our findings support previous PVH and BNST noradrenergic lesion studies, future studies are required to dissect the contribution of other co-expressed neuropeptides/neurotransmitters in mediating the feeding effects of NTS A2 neuron stimulation, as well as the necessity of NTS A2 neurons in food intake control. Attempts at inhibiting NTS A2 neurons using chemogenetics were unsuccessful due to the lack of specificity of the inhibitory designer receptor to NTS A2 neurons. This could be due to Cre recombination issues with TH Cre animal model or hM4Di transgene, and highlights the need to validate viral constructs and transgenic animal models. The physiological relevance of NTS A2 neurons remains to be determined and other loss of function studies are warranted. Collectively, this study demonstrates the sufficiency and functional heterogeneity of NTS A2 neurons in food intake control and highlights the importance for selective targeting of NTS A2 pathways to better understand the neural mechanisms of NTS A2 neurons in the control of feeding behaviors.

## Supplementary information

Supplementary material
