# Supplementary material for: Nucleus of the solitary tract A2 neurons control feeding behaviors via projections to the paraventricular hypothalamus
Source: Neuropsychopharmacology. 2022 Sep 16;48(2):351–61. doi: 10.1038/s41386-022-01448-5 (PMC9751294; doi:10.1038/s41386-022-01448-5)
Supplement: Supplementary file 1 — Supplementary material [file 41386_2022_1448_MOESM1_ESM.docx]

Supplementary Materials and Methods

**Stereotaxic surgery**

Rats received subcutaneous injection of atropine and were anaesthetized with 1.3 mL/kg Ketamine (Ketamil 100 mg/mL, Ilium) and 0.3 mL/kg Xylazine (Xylazil 20 mg/mL, Ilium). Once anaesthetized, rats were placed on stereotaxic frame and stereotaxic surgeries were performed for viral infusions and cannula implantations. Cannulae were secured with jeweler’s screws and dental cement. Post-surgery, rats received subcutaneous injections of analgesic Carprofen (Rimadyl 3 mg/kg) and antibiotics (Duplocillin 0.15 mL/kg). Rats were monitored daily for 7 days after surgery before commencement of experiments.

**Viral vectors and infusions**

Viral vectors were obtained as a gift from Bryan Roth through Addgene. pAAV-hSyn-DIO-eGFP (titre: 4.1 x 10^12^ vg/mL; Addgene viral prep #50457-AAV5; <http://n2t.net/addgene:50457>; RRID: Addgene_50457), pAAV-hSyn-DIO-hM3Dq (titre: 7.7 x 10^11^ vg/mL; Addgene viral prep #44361-AAV5; <http://n2t.net/addgene:44361>; RRID: Addgene_44361), pAAV5-hsyn-DIO-hM4Di (titre: 3.2 x 10^11^ vg/mL; Addgene plasmid # 50475 ; http://n2t.net/addgene:50475; RRID:Addgene_50475; virus packaged by Vector and Genome Engineering Facility, Children’s Medical Research Institute, NSW Australia), pAAV-hsyn-DIO-hM4Di (titre: 2.2 x 10^13^ vg/mL; #50475-AAV9; http://n2t.net/addgene:50475; RRID:Addgene_50475), pAAV-hsyn-DIO-mcherry (titre: 2.1 x 10^13^ vg/mL; Addgene viral prep # 50459-AAV9; http://n2t.net/addgene:50459; RRID:Addgene_50459). Viral vectors were infused at a rate of 100 nL/min using a 26 GA 5 µL syringe attached to a syringe pump (World Precision Instruments, FL, USA). The needle was left in place for a further 2 min to ensure complete virus infusion.

**Stereotaxic coordinates**

NTS coordinates were chosen to target A2 neurons located at the caudal NTS at the level of the area postrema. NTS (AP: +1.9 mm from occipital, ML: ±0.5 mm, DV: -9.3 mm, 15° anterior-posterior angle), PVH (AP: -1.8 mm, ML: ±0.5 mm, DV: -7.7 mm), BNST (AP: -0.3 mm, ML: ±2.8 mm [10° angle towards midline], DV: -8.0 mm), PBN (AP: +1.0 mm from Lambda, ML: ±2.0 mm, DV: -7.0 mm, 20° anterior-posterior angle).

**Perfusions**

For histological processing, rats were sacrificed with an intraperitoneal injection of sodium pentobarbital (100 mg/kg; Virbac), and transcardially perfused with prewash solution containing 0.9% saline, heparin (360 μl/L), and sodium nitrite (12.5 ml/L), followed by 4% paraformaldehyde. Brains were extracted, post fixed in 4% paraformaldehyde and transferred to 20% sucrose solution.

**Chromogenic immunohistochemistry**

Brain sections were washed in phosphate buffer and endogenous peroxidases quenched with 3% H_2_O_2_ in 50% ethanol. Sections were then blocked in 5% normal donkey serum and incubated in primary antibodies overnight. Primary antibodies used include rabbit anti-GFP (1:1000, Thermofisher Scientific; RRID: AB_221569), rabbit anti-oxytocin (1:10,000; Immunostar; RRID: AB_572258), rabbit anti-CRF (1:10,000, Peninsula Laboratories; RRID: AB_518252) and goat anti-c-fos (1:1,000, Santa Cruz; RRID: AB_2629503). Sections were washed and incubated in secondary antibodies (1:500, Jackson Immunoresearch; RRID: AB_2340383, AB_2340584) followed by avidin-biotin binding (Vector laboratories) and reaction with diaminobenzidine (DAB) in acetate buffer (pH 6.0) to produce a brown substrate. For c-Fos labelling, nickel-DAB was used to generate a black product to distinguish from the brown OT or CRF labelling. Sections were mounted onto gelatin-coated slides. The following day, they were dehydrated in alcohol and histolene, and coverslipped with entellan (Sigma Aldrich, USA). Slides were scanned and images visualized using Axio scan slide scanner (Zeiss, Germany).

Quantification of cells immunopositive for oxytocin and c-Fos, as well as CRF and c-Fos were performed using Zen2 (Zeiss) imaging software by an experimenter blinded to treatment conditions.

**Fluorescent immunohistochemistry**

Brain sections were washed in phosphate buffer saline (PBS), blocked in 5% normal donkey serum and incubated in primary antibodies overnight. Primary antibodies used include sheep/rabbit anti-TH (1:1000, Merck Pty Ltd; RRID: AB_390204, AB_90755), rabbit anti-GFP (1:1000, Thermofisher Scientific; RRID: AB_221569) and rabbit/goat anti-c-fos (1:1,000, Santa Cruz; RRID: AB_2106783, AB_2629503). The next day, sections were incubated in secondary antibodies (1:500, Thermofisher Scientific; RRID: AB_2535792, AB_2534102, AB_141637, AB_2534105) for 2 hours, after which, sections were again washed in PBS and mounted. Antifade mountant, Permafluor (Thermofisher Scientific) was applied and coverslipped. Sections were visualized under a fluorescent microscope (Olympus BX53).

**Chemogenetic inhibition of NTS A2 neurons and CCK food intake test**

To validate and select a suitable inhibitory designer receptor, hM4Di, TH Cre rats were injected to the NTS, one of two Cre-dependent AAV serotypes encoding hM4Di: AAV5 (n=3) or AAV9 (n=5). Quantification for hM4Di (mcherry) and TH was performed as described in ‘**Validation of TH Cre rat model to target NTS A2 neurons**’. Given that we had already quantified the expression of AAV5 reporter control (Figure 1A), here, we only analysed NTS sections from TH Cre rats injected with the AAV9 reporter control (n=3).

For behavioural experiments, TH Cre rats were injected with AAV9-hsyn-DIO-hM4Di (n=5) or mcherry (n=6) to the NTS. Food was removed 2 h prior to start of test. Individually housed rats with NTS hM4Di (or mcherry control) received IP injections of either Vehicle or CNO (5 mg/kg) 45 min before Vehicle or CCK (5 µg/kg) injections. Immediately after Vehicle or CCK injections, chow was returned and chow intake measured at 0.5 h.

Supplementary results

**Inhibition of NTS A2 neurons attenuates the intake suppressive effects of CCK**

Quantification of hM4Di (mcherry) and TH expression in the NTS revealed that only 28% (AAV5) and 32% (AAV9) of hM4Di mcherry-labelled cells were TH-positive. The reporter control, mcherry showed 67% colocalization with TH (Supplementary Figure 1A).

Given the slightly higher co-expression of hM4Di and TH in AAV9 (vs AAV5), the AAV9 serotype was used to examine whether TH Cre rats injected with these viral vectors exhibit feeding behaviors consistent with the role of NTS A2 neurons. Previous studies show that lesioning NTS A2 neurons block the effects of CCK [5]. Thus, we examined whether chemogenetic inhibition of NTS A2 neurons attenuates the intake inhibitory effects of CCK. Results showed a treatment 1 (Vehicle/CNO) x treatment 2 (Vehicle/CCK) x genotype interaction (F_(1,9)_=5.728, *P*<0.05). Separate analysis showed that while there was a main effect of CCK in both hM4Di (F_(1,4)_=11.025, *P*<0.05) and mcherry rats (F_(1,5)_=42.805, *P*<0.01), there was a treatment 1 x treatment 2 interaction only in hM4Di rats (F_(1,4)_=8.139, *P*<0.05). We followed up this interaction with pairwise analysis to show that chow intake in CNO/CCK treatment was significantly higher than chow intake in Vehicle/CCK treatment (t=3.243, *P*<0.05), and not different from Vehicle/Vehicle or CNO/Vehicle (Supplementary Figure 1B). Thus, consistent with past work, our results show that CNO significantly attenuated the intake suppressive effects of CCK.

Supplementary Figure 1


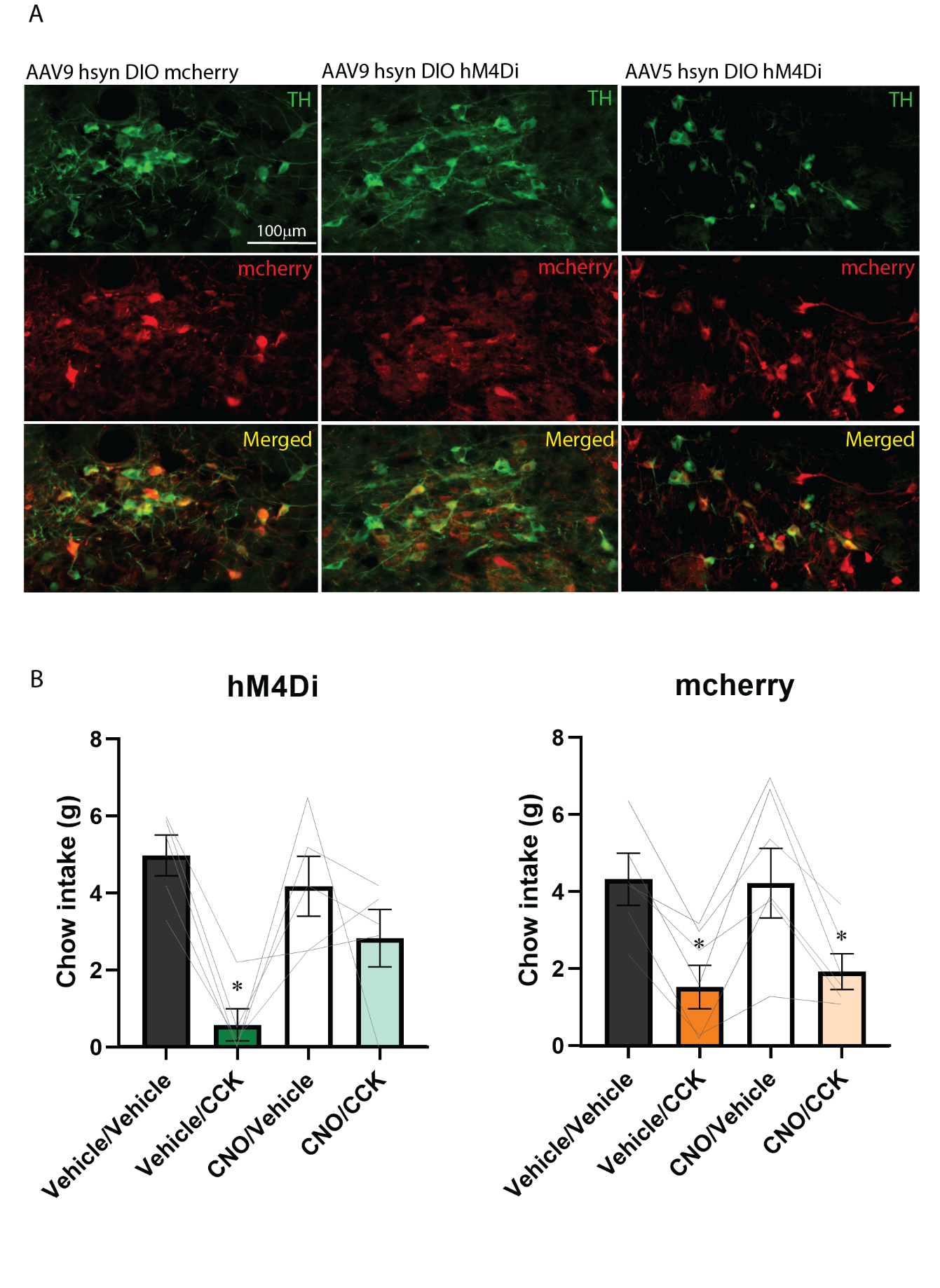


Supplementary Figure 1: (A) NTS hM4Di/mcherry expression after infusion of AAV9-hsyn-DIO-mcherry, AAV9-hsyn-DIO-hM4Di or AAV5-hsyn-DIO-hM4Di and co-localisation with TH. (B) Effects of CNO on CCK-induced suppression of chow intake in TH Cre rats injected with AAV9-hsyn-DIO-hM4Di or AAV9-hsyn-DIO-mcherry to the NTS. * *P*<0.05 compared to Vehicle.

Supplementary Tables

Supplementary Table 1: Average number of TH and fluorogold labelled cells in the NTS

|  | TH | FG | TH+FG | TH+FG % TH | TH+FG  % FG |
| --- | --- | --- | --- | --- | --- |
| PVH | 25.3±0.1 | 5.0±0.5 | 2.9±0.1 | 12.3±0.3 | 55.3±1.7 |
| BNST | 19.2±0.2 | 11.2±2.1 | 5.1±0.2 | 24.6±0.7 | 46.4±4.6 |

Supplmentary Table 2: Analysis of c-Fos, OT, CRF immunoreactivity in PVH following activation of NTS A2 neurons with 1 mg/kg CNO vs Vehicle

|  | c-Fos ^#^ | c-Fos+OT | c-Fos+CRF | % c-Fos in OT cells | % c-Fos in CRF cells |
| --- | --- | --- | --- | --- | --- |
| Vehicle | 89.1±31.7 | 3.8±1.0 | 14.8±12.5 | 4.5±1.2 | 10.3±7.9 |
| CNO | 153.1±45.5 | 24.5±7.2 * | 61.8±22.4 | 14.1±2.5 * | 45.8±10.5 * |

**P*<0.05 Vehicle vs CNO

^#^ values are averaged across 2 series of PVH sections (Series 1: OT and c-Fos, Series 2: CRF and c-Fos)
